# Wild strains reveal natural variation in *C. elegans* avoidance behaviors

**DOI:** 10.1093/g3journal/jkaf243

**Published:** 2025-10-10

**Authors:** Emily A Polk, Alber Aqil, J B Collins, Robyn E Tanny, Erik C Andersen, Omer Gokcumen, Denise M Ferkey

**Affiliations:** Department of Biological Sciences, University at Buffalo, The State University of New York, Buffalo, NY 14260, United States; Department of Biological Sciences, University at Buffalo, The State University of New York, Buffalo, NY 14260, United States; Department of Biology, Johns Hopkins University, Baltimore, MD 21218, United States; Department of Biology, Johns Hopkins University, Baltimore, MD 21218, United States; Department of Biology, Johns Hopkins University, Baltimore, MD 21218, United States; Department of Biological Sciences, University at Buffalo, The State University of New York, Buffalo, NY 14260, United States; Department of Biological Sciences, University at Buffalo, The State University of New York, Buffalo, NY 14260, United States

**Keywords:** *C. elegans;* wild strain, nociception, avoidance, behavior, GWAS, WormBase

## Abstract

Chemical stimuli, including odorants and tastants, provide information about food availability and allow animals to avoid harmful environments. Over 6 decades of research using primarily 1 laboratory-adapted strain of the nematode *Caenorhabditis elegans* has yielded a wealth of information about how this animal senses and responds to chemical cues to survive. However, it was not known whether the chemosensory behavioral profile of this strain (named N2) is representative of the species. Using a collection of hundreds of wild *C. elegans* strains collected from around the globe, we assessed their abilities to respond to 3 aversive stimuli (the bitter tastant quinine, the heavy metal copper, and the detergent SDS) in a laboratory setting and found ∼10- to 20-fold differences in response sensitivities among the strains. Further, response sensitivities to one stimulus were largely uncoupled from responses to the other stimuli and uncorrelated with the geographical locations from which the wild strains were collected. Using genome-wide association studies, we identified unique regions significantly correlated with different responses to each stimulus. Near-isogenic lines were created to confirm the effects of 2 genomic regions on differential avoidance behavior to the bitter tastant quinine. Combined, we report remarkable natural variation in chemosensory behavioral responses among wild *C. elegans* strains and describe 2 new quantitative trait loci associated with decreased response sensitivity to quinine.

## Introduction

Within every species, there exists natural variation for biological traits (Alonso-Blanco et al. 2009; Wohlbach et al. 2014; Niepoth and Bendesky 2020). Powerful genomic approaches have allowed progress in understanding the genetic basis of traits such as organismal morphology, physiology, and even disease risk (Stulp and Barrett 2016; Cano-Gamez and Trynka 2020). Variation in human height, for example, has been linked to thousands of single nucleotide polymorphisms (SNPs) in the genome, which can explain roughly 10 to 40% of height variation (Yengo et al. 2022). However, much less is known about how genes influence behavior (Niepoth and Bendesky 2020), likely due in part to the complexity of behavior itself. Environmental cues must first be sensed and then integrated with internal states to generate context-appropriate responses. The neural circuits generating behavioral responses are also subject to modulation and remodeling, allowing animals to adapt to their surroundings.

Chemosensation (the combined senses of smell and taste) allows animals to detect and navigate toward a potential food source or mate while avoiding harmful/toxic environments and predation. However, much of what we know about these senses comes from the study of laboratory strains of animals. In the case of *Caenorhabditis elegans*, the isogenic laboratory-adapted strain named N2 was first isolated in 1951 from mushroom compost in Bristol, England (Brenner 1974; Sterken et al. 2015), and has been used for chemosensory experiments for over 60 years (Bargmann 2006; Ferkey et al. 2021). Little is known about how naturally occurring genetic variation among *C. elegans* wild strains affects chemosensory responses or how adaptive forces shape the response profiles of *C. elegans* populations throughout the world (Volkers et al. 2013; Glater et al. 2014; Crombie et al. 2022).

Beginning in the 1970s, systematic screens have been used to identify chemicals to which *C. elegans* responds in a laboratory setting (Dusenbery 1973; Ward 1973; Dusenbery 1974, 1975; Bargmann et al. 1993). Although initially providing a list of both attractive and aversive stimuli for experimental studies, more recent work has provided a better understanding of what many of these compounds might represent to the animal in its natural habitat. In the wild, *C. elegans* are typically found with decomposing fruits and other plant matter that provide environments rich in nutritious bacterial strains on which they feed (Frezal and Felix 2015). Volatile odorants given off by these bacteria likely provide long-range cues for *C. elegans* chemotaxis toward potential food sources (Samuel et al. 2016; Schulenburg and Felix 2017; Worthy et al. 2018). In contrast, *C. elegans* avoid many compounds that are harmful at high concentrations (Bargmann 2006; Ferkey et al. 2021), such as the bitter plant alkaloid quinine (Hilliard et al. 2004) and heavy metals such as copper (Bargmann et al. 1990; Sambongi et al. 1999). *C. elegans* also avoid the detergent SDS (Bargmann et al. 1990; Hilliard et al. 2002).

Detection of noxious stimuli (nociception) is evolutionarily important for animal survival, and impairments to the pain sensory system can lead to dysfunction throughout the body (Broom 2001; Sneddon 2015; Nesse and Schulkin 2019; Walters and Williams 2019). As the primary nociceptors in *C. elegans*, the 2 ASH sensory neurons are critical for the avoidance of most aversive stimuli, including volatile and soluble chemicals (including quinine, copper, and SDS), high osmolarity, and mechanosensory touch to the nose (Bargmann et al. 1990; Kaplan and Horvitz 1993; Troemel et al. 1995 ; Sambongi et al. 1999; Hilliard et al. 2002, 2004; Bargmann 2006; Liu et al. 2018; Ferkey et al. 2021). Self-protective avoidance responses are elicited following ASH activation as signals are relayed to downstream interneurons and motor neurons (White et al. 1986). Because they detect diverse types of stimuli, the ASH neurons are believed to be analogous to nociceptors that detect multiple pain modalities in systems ranging from other invertebrates such as *Drosophila* (Tracey et al. 2003; Zhong et al. 2010; Im and Galko 2012; Johnson and Carder 2012) to vertebrates (Besson and Chaouch 1987; Treede 1999; Lee et al. 2005). Although it has long been assumed that avoidance of N2-characterized chemicals correlates with adaptive pressures nematodes face in the wild, it is not actually known whether detection/sensitivity is universal among wild *C. elegans* isolated from different habitats.

A collection of more than 650 wild isolate strains of *C. elegans* has recently become available through the *Caenorhabditis* Natural Diversity Resource (CaeNDR, https://caendr.org; formerly the *C. elegans* Natural Diversity Resource, CeNDR), along with whole-genome sequence data and habitat isolation information for each strain, making genome-wide association (GWA) studies possible (Cook et al. 2017; Crombie et al. 2024). Using the wild strains and resources from CaeNDR, we investigated natural variation in aversive chemosensory responses to quinine, copper, and SDS. We found a considerable degree of natural variation in behavioral sensitivity to each stimulus, with responses between stimuli only weakly correlated. We also show that behavioral sensitivity to these aversive stimuli is not linked to a strain's geographical isolation location. Using genome-wide analysis, we provide evidence that distinct genomic regions are associated with differential behavioral sensitivity to quinine, copper, and SDS and present evidence of 2 new validated quantitative trail loci (QTL) for variable quinine responses.

## Materials and methods

### *C. elegans* culture

Wild strains and N2 used for behavioral analysis were maintained under standard conditions on NGMA (nematode growth medium containing 1% agar and 0.7% agarose) agar plates (Andersen et al. 2012) seeded with the OP50 strain of *Escherichia coli* bacteria (Brenner 1974). NGMA plates were used to minimize burrowing by the wild strains.

### Strains

For a list of strains used in this study, see Supplementary File 1. The first tab includes a list of all strains used in this study. The second tab lists the 234 strains used in the LOCO GWA mapping. The third tab lists the 145 strains used in the INBRED GWA mapping. In the second and third tabs, the boxes highlighted in green indicate the strains unique to the LOCO and INBRED mapping sets, respectively. The fourth tab shows the 134 strains shared between the 2 GWA mappings.

### Behavioral assays

Wild strains were freshly thawed, bleached to get rid of any contamination, and subsequently maintained for 3 generations at 20 °C on NGMA (Andersen et al. 2012). Control experiments indicated no significant differences between behavioral responses of animals grown on NGM vs NGMA plates. Soluble tastant assays were performed as previously described (Hilliard et al. 2002, 2004; Fukuto et al. 2004; Ezak et al. 2010; Krzyzanowski et al. 2013, 2016; Chaubey et al. 2023). Briefly, young adult *C. elegans* were moved from NGMA plates with OP50 to transfer plates without OP50 to remove residual bacteria and then to NGMA plates lacking the bacteria and allowed to acclimate “off food” for 20 min prior to assaying. A drop of the stimulus was placed in front of a forward-moving animal, and avoidance response was scored as the percentage of animals that initiated backward locomotion within 4 s of encountering the drop. Our assays used a “wet drop” that animals entered, rather than an absorbed “dry drop.” All behavioral assays were performed by the same researcher over the course of 2 months for quinine and copper and over 3 months for SDS. All assays were performed in duplicate (10 to 15 animals per plate), and each animal was tested only once. For wild strains, the average response of the 2 plates is reported (without relative calculations). N2 was included as the control for behavioral reproducibility each day, with the final N2 data representing the average of all N2 plates assayed in parallel to the wild strains. We found a ≤20% response difference between each assay plate for each strain. Tastants were dissolved in M13 buffer, pH 7.4 (Wood 1988). Strains tested for behavior following outcrossing to the N2 laboratory strain followed the same protocol as above but were maintained and assayed on NGM plates (Brenner 1974). The average behavioral response for each wild strain to all 3 stimuli is included in Supplementary File 2.

### Correlations

Correlation graphs were plotted in *R* using the package ggplot2 with the line of best fit setting (Wickham 2016). Correlation statistics and the *R*^2^ and *P* values for each stimulus comparison, were calculated in *R* using the summary statistics in base *R*.

### GWA studies

To test for correlations, GWA analyses were performed using the CaeNDR “Genetic Mapping” tool (https://caendr.org), as previously described (Cook et al. 2017; Zdraljevic et al. 2019; Lee et al. 2021; Widmayer et al. 2022). To minimize the effect of the high variability in Hawaiian genomes, subsets of wild strains excluding Hawaiian strains were used. The subset of 234 strains had none from Hawaii, and the set with 145 strains had only 2 Hawaiian strains. To correct for population structure, leave-one-chromosome-out (LOCO) excludes markers on the chromosome being left out, and associations are determined from the remaining chromosome markers. The INBRED association mapping uses a genomic relatedness matrix made specifically for organisms that are inbred (such as *C. elegans*) and corrected for genetic stratification. Both methods correct for individuals' relatedness between populations (Crombie et al. 2024). Associations were visualized using Manhattan plots. For full GWA results with test statistics, see Supplementary File 3 (LOCO) and Supplementary File 4 (INBRED).

### Near-isogenic lines

Near-isogenic lines (NILs) were made following the protocol as previously described (Zdraljevic et al. 2017, 2019; Evans et al. 2018; Nyaanga and Andersen 2022). NILs were created using a wild strain with a common haplotype crossed with N2 animals. After the initial crosses, only animals containing the desired crossover were kept for further outcrossing. Primer sets were designed such that 1 pair was able to detect the presence or absence of a deletion within the desired QTL, and the other pair detected the presence or absence of a deletion outside the desired QTL. This allowed us to maintain the desired genotype of the QTL region as well as the alternative parent strain for the rest of the genome. These animals were then backcrossed 3 more times, ensuring the desired genotype for the region of interest was retained. After strains were backcrossed 4 times, they were whole-genome sequenced (Zdraljevic et al. 2017, 2019; Evans et al. 2018). Other regions of donor strain sequence were present in other spots of the genome, but for NIC515>N2, JU2593>N2, and N2>JU2593, these regions were not shared between NILs. For the 2 N2>NIC515 NIL lines, 1 small shared region of N2 genomic DNA was outside the QTL (position 1 to 1,524,726 of chromosome IV, which corresponds to 8.7% of the chromosome). However, neither the LOCO nor INBRED GWA analyses revealed a significant peak in this region.

### Maps

The geographical maps were made using the *R* package rworldmap, with the mapBubbles function (South 2011). The mapRegion setting was adjusted to show only Europe. The specific location from which each strain was collected (latitude and longitude) was obtained from CaeNDR, and the continental data were extracted based on the country of origin listed.

## Results

### Response to aversive stimuli varies among *C. elegans* wild strains

**Fig. 1.**
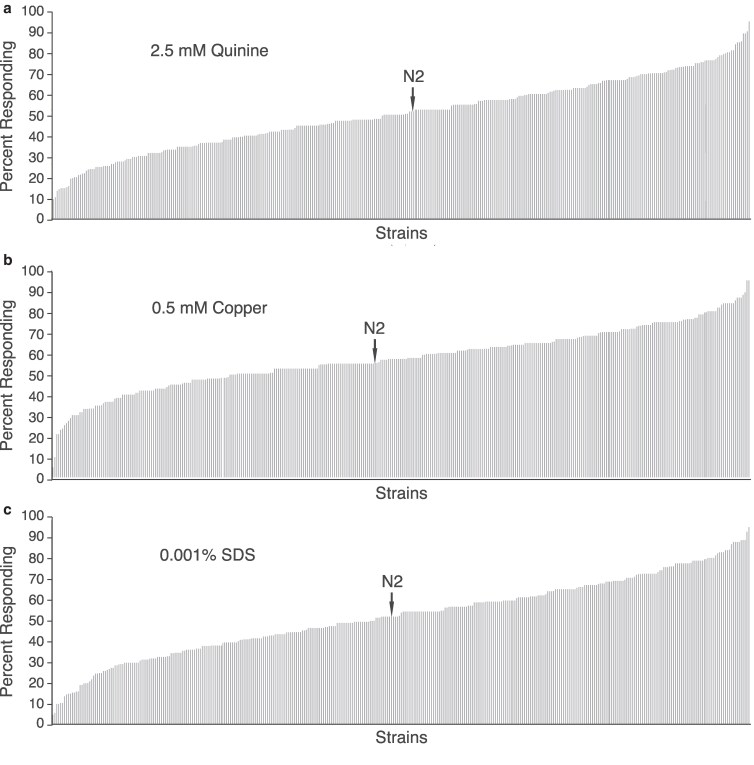
Variation in *C. elegans* wild strain responses to aversive stimuli. A broad range of behavioral sensitivity was observed across the 364 wild strains when assayed for avoidance of 2.5 mM quinine (a), 0.5 mM copper (b), and 0.001% SDS (c). In each case, the laboratory strain N2 responded to these concentrations ∼50% of the time. The percentages of animals responding are shown. Data represent the average of behavioral experiments performed in duplicate for each wild strain.

The laboratory N2 strain of *C. elegans* responds to and avoids a broad range of aversive chemical stimuli (Bargmann 2006; Ferkey et al. 2021). However, it is not known whether the degree of nociceptive sensitivity observed for N2 is representative of *C. elegans* response in general. To investigate the extent of natural variation in aversive behavior among *C. elegans*, 364 wild isolate strains were obtained from the *Caenorhabditis* Natural Diversity Resource (CaeNDR) (Cook et al. 2017; Crombie et al. 2024) and assayed for their behavioral responses to 3 aversive stimuli commonly used in laboratory experiments: quinine, copper, and SDS (Bargmann 2006; Ferkey et al. 2021). To ensure that both decreased and increased behavioral sensitivity relative to the common laboratory strain could be assessed, the concentration of each stimulus to which N2 reproducibly responded ∼50% of the time was used (2.5 mM quinine, 0.5 mM copper, and 0.001% SDS). We observed a remarkable degree of behavioral variation among the wild strains for all 3 stimuli, with response rates ranging from ∼9 to 90% for quinine, ∼5 to 90% for copper, and ∼4 to 88% for SDS (Fig. 1). Furthermore, in each case, N2 was roughly centered along the *x*-axis, with a similar proportion of wild strains showing decreased vs increased responses relative to N2 (Fig. 1). These data reveal a large degree of variation in nociceptive sensitivity among *C. elegans* wild strains.

### Response to one stimulus does not strongly predict response to another

**Fig. 2.**
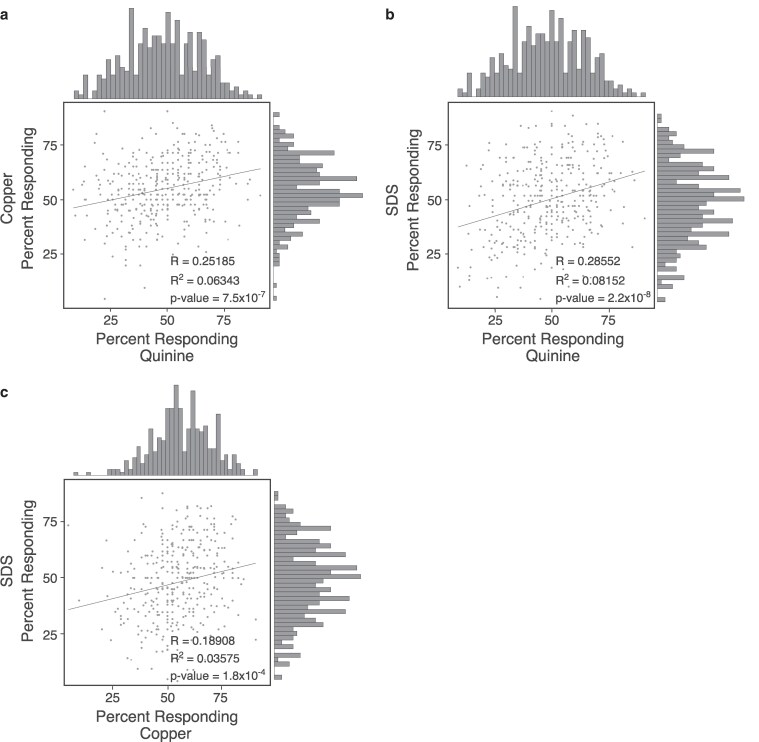
Behavioral correlations between quinine, copper, and SDS. Correlations of the 364 wild strain responses between quinine and copper (a), quinine and SDS (b), and copper and SDS (c) are shown. Each point represents an individual strain's response to 1 stimulus plotted along the *x*-axis and its response to the second stimulus plotted along the *y*-axis. Graphed along each correlation plot are the number of strains with a given average response to each individual stimulus in a bin size of 2%. The line of best fit along with the *R* and *R*^2^ values and the *P* values are shown for each comparison. We considered *R* < 0.3 as weakly correlated (Cohen 1988).

Quinine, copper, and SDS are each thought to be detected by unique receptors and signal through distinct signal transduction cascades, although some downstream signaling components could be shared (Bargmann 2006; Ferkey et al. 2021). Thus, genetic variation in genes unique to a particular stimulus would be unlikely to affect response to another stimulus. By contrast, variation in signaling components or regulatory molecules shared by more than 1 signal transduction cascade could alter responses to multiple stimuli. To determine if a wild strain's behavioral response to one aversive stimulus is correlated with its response to another of the aversive stimuli tested, each pairwise combination was plotted: quinine to copper (Fig. 2a), quinine to SDS (Fig. 2b), and copper to SDS (Fig. 2c). The correlation between the responses to different stimuli are weak (*R* < 0.286, *R*^2^ < 0.082) but not random (*P* < 0.05). In other words, the response of many wild strains to one stimulus is not the same as its response to another stimulus (see also Supplementary File 2), suggesting that the genetic factors underlying the variation in *C. elegans* avoidance behaviors likely affect different signaling or regulatory molecules for each stimulus.

In addition to directly plotting the response correlations, response frequencies were binned (each bar representing a bin size of 2%) and graphed along the plots to reveal the relative distribution of response frequency for each stimulus. For all 3 stimuli, the number of strains responding modeled a normal distribution (Fig. 2). A normal distribution is expected when the traits are regulated by multiple genetic and environmental factors, as is seen in virtually all complex traits in humans and multiple model organisms (McCarthy et al. 2008). Given that the environment is controlled in the laboratory setting, our results suggest that the majority of the observed behavioral variation has a yet-to-be-understood polygenic basis.

### GWA analysis revealed unique peaks for each stimulus

**Fig. 3.**
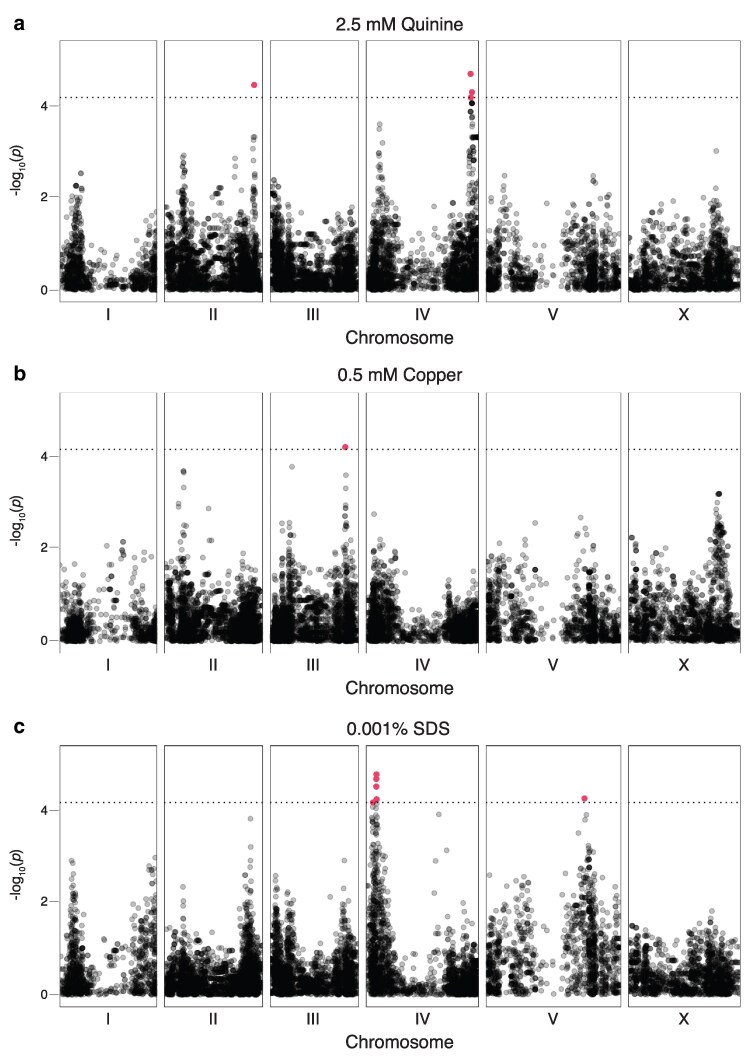
GWA for 3 aversive stimuli. Significant QTL were identified from the LOCO genome-wide association mapping (234 strains, no Hawaiian) for quinine (a), copper (b), and SDS (c). The *x*-axis displays the chromosome position of the variants, and the *y*-axis shows the significance of each variant. In each Manhattan plot, chromosomes I–V and X are shown. The horizontal dashed lines indicate the EIGEN threshold of significance in each plot (Zdraljevic et al. 2019). Each point represents 1 SNV, identified from the variant data from CaeNDR (20231213 release) (Cook et al. 2017; Crombie et al. 2024). Red points indicate significant SNVs.

To understand the genetic basis of behavioral variation, we used the wild strain whole-genome variant data available from CaeNDR (20231213 release), in conjunction with our behavioral data (Fig. 1), to perform GWA analyses for quinine, copper, and SDS. This analysis sought the identification of haplotypes that are significantly correlated with varied behavioral response to each stimulus (Fig. 3a–c). LOCO association mapping was used first to correct for population structure, using a subset of the wild strains to remove the effect of the high genomic variability among strains isolated from Hawaii (234 strains included, no Hawaiian; see Supplementary File 1) (Widmayer et al. 2022). We note that the ends of *C. elegans* autosomal chromosome arms are hyperdivergent, which likely accounts for the “U” shape seen for individual chromosomes in the Manhattan plots and might indicate balancing selection (Lee et al. 2021). Using this approach, we identified 1 robust peak on the right arm of chromosome IV (referred to as IV-R) significantly correlated with differences in behavioral avoidance of quinine (Fig. 3a). There was 1 additional peak on the right arm of chromosome II, represented by only 1 significant single nucleotide variant (SNV) (Fig. 3a). One peak was associated with differential responses to copper (right arm of III, Fig. 3b), and 2 were associated with responses to SDS (1 on the left arm of IV referred to as IV-L and 1 on the right arm of V, Fig. 3c).

To further correct for relatedness between populations, a second analysis was performed using INBRED association mapping and a smaller subset of 145 strains (only 2 Hawaiian; see Supplementary File 1) (Supplementary Fig. 1). This approach revealed a robust peak on the left arm of chromosome II (referred to as II-L) correlated with differences in quinine response, along with a peak on the right arm of II (that did not overlap with the peak identified using LOCO, Fig. 3a) and 1 on the left arm of chromosome V represented by 1 SNV (Supplementary Supplemental Fig. 1a). For copper and SDS response, peaks represented by single SNVs were seen on chromosome III and X, respectively (Supplementary Fig. 1b and c). Notably, none of the significant SNVs from each stimulus were found in the other stimuli. Combined, these data suggest that variation in response sensitivity to each stimulus has a largely unique genetic basis.

To determine if the most significant peaks from the GWA studies (II-L and IV-R for quinine, IV-L for SDS) were associated with increased vs decreased behavioral sensitivity, the most significant SNV from each was plotted for comparison. Because *C. elegans* is a self-fertilizing species, all SNVs are assumed to be homozygous in the wild strains maintained and distributed by CaeNDR (Frezal and Felix 2015; Cook et al. 2017; Crombie et al. 2024). Wild strains with the alternate SNV on chromosome II-L at position 2,792,986, or the alternate SNV on chromosome IV-R at position 16,214,422, displayed a decreased behavioral sensitivity to quinine (58% vs 44% responding and 50% vs 33% responding, respectively; Supplementary Fig. 2a and b). Strains with the alternate SNV at position 1,689,139 of chromosome IV-L had a slightly elevated behavioral response to SDS (48% vs 59% responding; Supplementary Fig. 2c). Taken together, we found that distinct genomic regions are associated with differential responses to each aversive stimulus, consistent with the lack of strong correlation between responses to individual stimuli (Fig. 2).

### Two QTL underlie differences in behavioral sensitivity to quinine

**Fig. 4.**
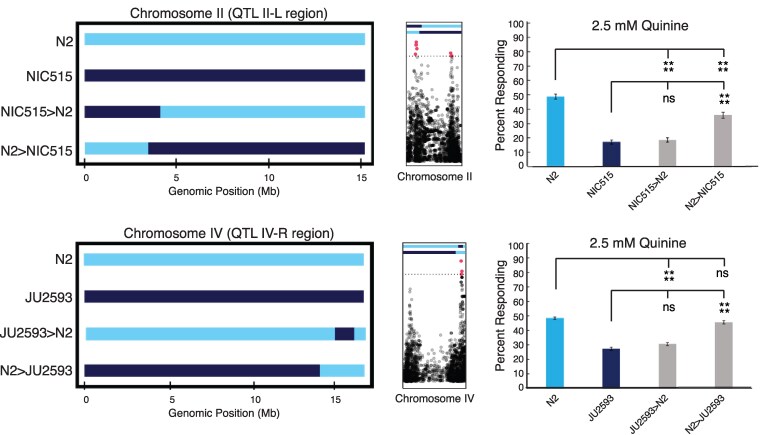
Response of NILs to 2.5 mM quinine. NILs were made for the significant quinine response peaks on the left arm of chromosome II (II-L) and right arm of chromosome IV (IV-R). For each region, NILs were made such that some had the N2 genome background with the wild isolate sequence in the QTL only, and the reciprocal, strains containing the wild strain genome background with the N2 sequence in the same QTL. NIC515>N2 and JU2593>N2 indicate wild strain QTL introgressed into the N2 genomic background. N2>NIC515 and N2>JU2593 indicate N2 QTL introgressed into the respective wild strain genomic backgrounds. The size of QTL shown is based on only the shared genomic regions of at least 2 NIL strains' whole-genome sequence data for each pair of NILs representing the QTL, with N2 genomic sequence shown in light blue and wild strain sequence in dark blue. Only the genomic regions shared across NIL lines are indicated. See Materials and methods for additional information. The center panels show the position of the NIL regions relative to the GWA peaks for QTL II-L and IV-R. The corresponding behavioral responses of N2 (laboratory strain, light blue), the corresponding wild strains (dark blue), and complementary NILs (light gray bars) are shown in the panels on the right. Each behavioral response bar shows the pooled data of at least 2 NILs and ≥ 120 animals tested over 3 days. The error bars indicate the standard error of the mean (SEM). Each NIL type was pooled (eg all lines with wild strain QTL in N2 background for chromosome II), and ANOVA tests were performed for significance. *P < 0.05, **P < 0.01, ***P < 0.001, and ****P < 0.0001. ns denotes *P* ≥ 0.05 (not significant).

To test the causality of differential behavior of the 2 most significant peaks from the quinine GWA mappings (II-L and IV-R; Supplementary Fig. 1a and Table 1 and Fig. 3a,), NILs were created and assayed (Fig. 4). Following a mating between 2 different *C. elegans* strains, subsequent self-fertilization by hermaphrodites leads to megabase-sized haplotype blocks containing hundreds of SNVs, making it challenging to identify individual causal nucleotide variants within GWA QTL (Andersen and Rockman 2022; Widmayer et al. 2022). With this limitation in mind, we tested entire haplotype blocks for their contributions to behavioral variation, rather than individual variants within haplotype blocks.

For both, when the wild strain QTL was swapped into the N2 genomic background (NIC515>N2 and JU2593>N2), diminished quinine sensitivity was observed, with responses similar to the corresponding wild strain (Fig. 4). Additionally, in each case, the QTL tested explained at least 84% of the difference in behavior between N2 and the corresponding wild strain (Supplementary Table 1). However, when the reciprocal NILs were analyzed, with the N2 regions swapped into the wild strain genomic backgrounds, only lines with the N2 QTL on chromosome IV-R (N2>JU2593) responded similar to N2 animals (Fig. 4), with 86% of the difference in behavior explained by this region (Supplementary Table 1). When the II-L N2 region was swapped into the wild strain background (N2>NIC515), response sensitivity was more than the wild strain but did not fully reach the response of N2 (Fig. 4). This QTL also had a lower explained difference (58%) when analyzed in this direction (Supplementary Table 1). To confirm the specificity of these QTL for variable quinine response, the NILs were also tested in parallel for response to SDS. In both cases, the genomic background (not the swapped QTL) dictated response to this ASH-detected stimulus (Supplementary Fig. 3). Taken together, we have identified 2 QTL representing common alternative haplotypes that are responsible for decreased quinine sensitivity among wild strains compared to the laboratory N2 strain.

## Discussion

Since its isolation over 70 years ago, the N2 strain of *C. elegans* has been used for nearly all studies of the species' development, physiology, and behavior—with the longstanding assumption that this strain is representative of the species at large. Although some differences between individual strains have been reported (Gaertner and Phillips 2010), including in some chemosensory behaviors (Volkers et al. 2013; Glater et al. 2014; Crombie et al. 2022; Lansdon et al. 2022; Amoroso et al. 2024), only recently have large-scale comparisons between N2 and additional *C. elegans* strains become possible due to the large wild strain collection now available through CaeNDR (Cook et al. 2017; Evans et al. 2021; Andersen and Rockman 2022; Crombie et al. 2024). Thus, the study of within-species variation of traits has emerged as a new frontier to both investigate the genetic basis of biological variation in *C. elegans* and recalibrate the role of N2 as the reference laboratory strain as we seek to understand the biology of this nematode.

Across diverse species, an animal's senses of smell and taste help it to find desirable and nutritive food sources. These senses are also critical for rejecting spoiled or potentially poisonous substances. Evidence from nonhuman species suggests that taste has been shaped by evolution, likely reflective of food availability and diet (Reed and Knaapila 2010; Pajic et al. 2019). For example, animals such as cats and chickens have lost the function of their sweet receptor, likely because the foods they eat contain little sugar (Li et al. 2005; Shi and Zhang 2006; Reed and Knaapila 2010). However, it is possible that causality could go in the other direction. Humans display significant person-to-person differences in the ability to smell and taste (Blakeslee and Salmon 1935; Menashe et al. 2003; Reed and Knaapila 2010; Newbomb et al. 2012). Although the ability to smell some odorants [eg cinnamon (Knaapila et al. 2007)] is heritable, little or no heritability is associated with others [eg chocolate, lemon (Hubert et al. 1980; Knaapila et al. 2008)]. Human twin studies (comparing monozygotic and dizygotic twin pairs) have also suggested that the perception of many smell and taste stimuli are at least partially determined by genotype, although environmental factors could also contribute (Knaapila et al. 2012).

The complex natural environment of *C. elegans* necessitates that these animals be able to sense and respond robustly and sensitively to a range of chemical cues for optimal survival and reproduction (Bargmann 2006; Ferkey et al. 2021). To understand the extent to which the behavior of the N2 laboratory strain represents the behavior of *C. elegans*, we tested hundreds of wild strains collected from around the globe (every continent except Antarctica) for their response to 3 aversive stimuli, all detected primarily by the ASH nociceptive neurons: the bitter tastant quinine, the heavy metal copper, and the detergent SDS. We observed a remarkable degree of variation in behavioral sensitivity to each, with wild isolate strains ranging from severely hyposensitive to extremely hypersensitive and N2 representing the approximate median of response at the concentrations tested (Fig. 1). Given that nociception is a protective behavioral response to potentially harmful stimuli, it was particularly unexpected that so many strains had decreased sensitivity compared to the N2 strain. Because response sensitivities were not strongly correlated between stimuli, it also suggests that distinct genetic differences working through different mechanisms and not shared components of the signal transduction pathways likely underlie the observed behavioral variation. For example, variation in pheromone receptor genes underlies differences in pheromone sensitivity among wild strains (Lee et al. 2019).

To assess the relationship between sensory behavior and the ecology of the environments from which *C. elegans* strains were isolated, we ran regression analyses using all available habitat ecological variables, detailed in CaeNDR and prior work (Cook et al. 2017; Evans et al. 2017; Crombie et al. 2024), for quinine, copper, and SDS. We did not find any that strongly predicted response sensitivities to the 3 aversive stimuli. However, it is important to note that it is plausible that within-region population structure in this self-fertilizing species might be much stronger than many other species, generating local, rather than global, adaptive trends that would not be captured in our study. We also asked whether behavioral response sensitivities to quinine, copper, or SDS correlated with the geographical locations from which wild isolates originated, but we found no association when looking at the gross scale of country/continent (Supplementary Fig. 4). In each grouping, approximately equal proportions of hyposensitive, average and hypersensitive strains for response to quinine (Supplementary Fig. 4a), copper (Supplementary Fig. 4b), and SDS (Supplementary Fig. 4c) were found. When wild strains from the largest continental grouping—individuals from Europe—were plotted on a map (Cook et al. 2017; Crombie et al. 2024) and color coded by sensitivity, an intermixed distribution of hyposensitive, average, and hypersensitive strains was seen across countries for each stimulus (Supplementary Fig. 4, right side panels). Therefore, our study sets the stage for future work where sample collection is well contextualized, including at the local scale, with regard to the chemicals and bacteria present in the soil, as well as the presence or absence of possible predators. For example, differences in behavioral avoidance of the bacterial parasite *Serratia marcescens* were recently reported using a small subset (12) of genetically divergent *C. elegans* strains, and strains with higher avoidance also had higher fitness as measured by population growth rate (Amoroso et al. 2024).

Approaches such as genetic screens or the study of CRISPR-generated alleles in model organisms can lead to the identification and characterization of genes involved in a biological process of interest, including behavior, but many times they cause phenotypes that would severely impact survival in the wild. Thus, they are likely not representative of the genetic variation that exists or can persist in nature. Furthermore, although traditional forward genetic screens with the N2 strain have identified several components of *C. elegans* chemosensory signal transduction cascades (Bargmann 2006; Ferkey et al. 2021), such screens are limited in scope as to what they are likely to uncover. In general, only mutants at the extremes of a response (eg profoundly defective relative to controls) can be identified in a high-throughput manner or mapped using traditional approaches. Mutations in genes that cause more subtle changes in sensitivity are missed, as are alleles that influence multigenic complex traits.

To understand the impact of the genome on aversive behavioral differences, we performed GWA analyses and found significant peaks for each stimulus (Fig. 3 and Supplementary Fig. 1). We used subsets of the wild strains (Supplementary File 1) to minimize bias in population structure, following best practices in *C. elegans* GWA studies (Widmayer et al. 2022). We note that only 134 wild strains were common to the 2 GWA mappings. Although the INBRED mapping set included only 11 unique strains, the larger LOCO mapping set included 100 strains that were not part of the INBRED mapping. The inclusion of these additional strains likely accounts for the differences in regions identified. We verified our findings using NILs to directly test the role of 2 significant regions identified by GWA for their role in quinine response (Fig. 4). Wild strains with the most common alternative haplotype in the QTL represented by the most significant SNV were chosen to generate the NILs. We found that both QTL contribute to differences in quinine sensitivity, and when either wild strain QTL was crossed into the N2 background, it was sufficient to dampen the response of the laboratory strain to a level comparable to that of the parent wild strain, while crossing the N2 QTL into the wild strain background elevated behavioral response (Fig. 4). In contrast, these QTL do not underlie response to an alternate ASH-detected stimulus (SDS; Supplementary Fig. 3).

In our 2 confirmed QTL, we identified over 300 genes annotated in the N2 reference genome that contain variants that could putatively affect function (Supplementary Tables 2 and 3), not including additional/alternate genes present in the wild strains. Among these, genes encoding G protein-coupled receptors (GPCRs) are highly represented (12% of II-L, 23% of IV-R) and could potentially function as chemosensory receptors. However, diverse gene categories are represented in both QTL, and over 40% of the annotated genes remain unnamed. Further, noncoding variation in regulatory sequence could also account for phenotypic differences, and could in some cases even be more evolutionarily important (Ayyadevara et al. 2001, 2003; Vertino et al. 2011; Evans et al. 2021). Based on association studies in humans (van Rheenen et al. 2019; Abdellaoui and Verweij 2021), it is also likely that changes in multiple genes account for the differences in sensory response.

Across the *C. elegans* genome, there are 366 distinct regions that are hyperdiverged among isotypes (distinct genome-wide genotypes), representing ∼20% of the N2 reference genome (Lee et al. 2021). Interestingly, these hyperdivergent regions are enriched for environmental-response genes and discussed within the context of balancing selection, suggesting a role in helping animals to flourish in a range of environments (Lee et al. 2021). Although distributed throughout the genome, the left arm of chromosome II and the right arm of chromosome IV are highly enriched for hyperdiverse regions across isotypes (Lee et al. 2021). Using published data (Lee et al. 2021) and data available on CaeNDR, we compared the genome of the N2 laboratory strain to the genomes of the wild strains that were crossed to N2 to generate the NIL lines. We found 7 hyperdivergent regions in the II-L QTL (when comparing N2 with NIC515) ranging in size from 8 to 159 kb, and 5 in the IV-R QTL (when comparing N2 with JU2593) ranging in size from 5 to 24 kb. Thus, these hyperdivergent and adaptively evolving regions also could contribute to differences in behavior between strains, providing an exciting venue for future research.

Taken together, our study is the first of this scale to assess how natural genetic variation shapes *C. elegans* behavior. We found a surprising range in nociceptive sensitivity across the hundreds of wild strains assayed, identified unique loci correlated with differential responses to 3 aversive stimuli (quinine, copper, SDS), and confirmed the role of 2 distinct QTL (II-L and IV-R) in generating differences in quinine avoidance sensitivity. Future experiments will require generating NILs containing smaller QTL regions to further understand what specific change(s) within these QTL allow animals to respond differently to quinine. Ultimately, understanding the genetic basis for naturally occurring range of nociceptive sensitivity may provide new ways to manage aversion and pain across species.

## Supplementary Material

jkaf243_Supplementary_Data

## Data Availability

Plasmids and NIL strains are available upon request. All data necessary for confirming the conclusions of the article are present within the article, figures, tables, and supplemental files. Supplementary File 1 is a list of strains and their origin, with tabs denoting strain subsets. Supplementary File 2 contains the behavioral data. Supplementary File 3 is the LOCO GWA results with test statistics. Supplementary File 4 is the INBRED GWA results with test statistics. Supplemental material available at G3 online.
