# Supplementary material for: Wild strains reveal natural variation in C. elegans avoidance behaviors
Source: G3 (Bethesda). 2025 Oct 10;15(12):jkaf243. doi: 10.1093/g3journal/jkaf243 (PMC12693514; doi:10.1093/g3journal/jkaf243)
Supplement: jkaf243_Supplementary_Data [file jkaf243_supplementary_data.zip › Supplemental_Material_Legends_G3-2025-406145.docx]

**Supplemental Material Legends:**

**Supplemental Figure 1. Alternative GWA for quinine response**

Additional QTL were identified using the INBRED genome-wide association mapping (145 strains, 2 Hawaiian) for quinine (A), copper (B), and SDS (C). The x-axis displays the chromosome position of the variants, and the y-axis shows the significance of each variant. In each Manhattan plot, chromosomes I-V and X are shown. The horizontal dashed lines indicate the EIGEN threshold of significance in each plot (Zdraljevic *et al.* 2019). Each point represents one SNV, identified from the variant data from CaeNDR (20231213 release) (Cook *et al.* 2017; Crombie *et al.* 2024). Red points indicate significant SNVs.

**Supplemental Figure 2. Violin plots of GWAS peaks**

Using CaeNDR, violin plots were used to compare the behavioral response sensitivity of strains homozygous for the reference allele (Ref; includes the N2 strain) to strains with two copies of the alternative allele (Alt). The variants on chromosome II at nucleotide 2,792,986 (A) and chromosome IV at nucleotide 16,214,422 (B) are both associated with decreased behavioral response to quinine. The variant positioned on chromosome IV at nucleotide 1,689,139 is associated with slightly increased behavioral response to SDS (C).

**Supplemental Figure 3. Response of near-isogenic lines (NILs) to 0.001% SDS**

The same NIL lines shown in Figure 4 were also tested for response to SDS. Genomic content (N2 versus wild strain) is shown on the left. The center panels show the position of the NIL regions relative to the GWA peaks for QTL II-L and IV-R. The corresponding behavioral responses of N2 (laboratory strain, light blue), the corresponding wild strains (dark blue), and complementary NILs (light grey bars) are shown in the panels on the right. The combined data of ≥ 60 animals tested over three days are shown for each line. The error bars indicate the standard error of the mean (SEM). ANOVA tests were performed for significance. * denotes *p* < 0.05, ** denotes *p* < 0.01, *** denotes *p* < 0.001 and **** denotes *p* < 0.0001. ns denotes *p* ≥ 0.05 (not significant).

**Supplemental Figure 4. Geographical locations and behaviors of wild strains by continent**

Strains were binned by their behavior [hyposensitive (0-39.99%) shown in blue, average (40-60%) in yellow, hypersensitive (60.01-100%) in red] and sorted by the continent from where they were collected (separating Hawaii from North America) for behavioral response to quinine (A), copper (B), and SDS (C). Twelve strains had no known collection location, and these strains are labeled in the bar graph as “Unknown”. A map of the isolation locations within Europe, the continent with the largest total number of wild strains, is shown for each stimulus. Each colored circle corresponds to the latitude and longitude coordinates of one strain, and each was also colored to represent behavioral sensitivity as in the bar graph.

**Supplemental Table 1. Summary of quinine GWA peaks**

The base pair (bp) interval for the quinine GWA QTL on the left arm of chromosome II (II-L) and the QTL on the right arm of chromosome IV (IV-R) are given, along with the position of the peak SNV in each and the calculated effect size contribution of the QTL (percentage of the parental strain difference) recapitulated by the NILs.

**Supplemental Table 2. Annotated genes in quinine QTL II-L**

The annotated genes within the quinine QTL on the left arm of chromosome II of the N2 reference genome are listed, categorized as either unnamed, comprising multiple miscellaneous gene families, or predicted G protein-coupled receptors (GPCRs).

**Supplemental Table 3. Annotated genes in quinine QTL IV-R**

The annotated genes within the quinine QTL on the right arm of chromosome IV of the N2 reference genome are listed, categorized as either unnamed, comprising multiple miscellaneous gene families, or predicted G protein-coupled receptors (GPCRs).

**Supplemental File S1. List of strains used**

Tab 1 includes all strains used in this study. Tab 2 lists the strains used in the LOCO GWA mapping. Tab 3 lists the strains used in the INBRED GWA mapping. Boxes highlighted in green in Tab 1 and Tab 2 indicate the strains unique to each mapping. Tab 4 lists the strains common to the LOCO and INBRED mappings.

**Supplemental File S2. Behavioral assay data**

Each strain’s behavioral response to 2.5 mM quinine, 0.5 mM copper, and 0.001% SDS is shown, given as percent responding.

**Supplemental File S3. LOCO GWA**

The GWA results, with test statistics, for the LOCO genome-wide association mapping are shown.

**Supplemental File S4. INBRED GWA**

The GWA results, with test statistics, for the INBRED genome-wide association mapping are shown.

**Supplemental References:**

Cook, D. E., S. Zdraljevic, J. P. Roberts and E. C. Andersen, 2017 CeNDR, the *Caenorhabditis elegans* natural diversity resource. Nucleic Acids Res 45**:** D650-D657.

Crombie, T. A., R. McKeown, N. D. Moya, K. S. Evans, S. J. Widmayer *et al.*, 2024 CaeNDR, the *Caenorhabditis* Natural Diversity Resource. Nucleic Acids Res 52**:** D850-D858.

Zdraljevic, S., B. W. Fox, C. Strand, O. Panda, F. J. Tenjo *et al.*, 2019 Natural variation in *C. elegans* arsenic toxicity is explained by differences in branched chain amino acid metabolism. Elife 8.
