# Supplementary material for: Wild strains reveal natural variation in C. elegans avoidance behaviors
Source: G3 (Bethesda). 2025 Oct 10;15(12):jkaf243. doi: 10.1093/g3journal/jkaf243 (PMC12693514; doi:10.1093/g3journal/jkaf243)
Supplement: jkaf243_Supplementary_Data [file jkaf243_supplementary_data.zip › Supplemental_Table_1_G3-2025-406145.docx]

**Supplemental Table 1**

| **Trait** | **Chromosome** | **Interval (bp)** | **Peak SNV (bp)** | **Effect Size** |
| --- | --- | --- | --- | --- |
| Quinine | II | 1,602,322-3,549,168 | 2,792,986 | NIC515>N2 = 95%  N2>NIC515 = 58% |
| Quinine | IV | 15,612,478-16,789,401 | 16,214,422 | JU2593>N2 = 84%  N2>JU2593 = 86% |
