# Supplementary material for: Wild strains reveal natural variation in C. elegans avoidance behaviors
Source: G3 (Bethesda). 2025 Oct 10;15(12):jkaf243. doi: 10.1093/g3journal/jkaf243 (PMC12693514; doi:10.1093/g3journal/jkaf243)
Supplement: jkaf243_Supplementary_Data [file jkaf243_supplementary_data.zip › Supplemental_Table_2_G3-2025-406145.docx]

**Supplemental Table 2. Annotated genes in quinine QTL II-L**

| Unnamed genes | Named genes  (miscellaneous) | Named genes  (predicted GPCRs) |
| --- | --- | --- |
| B0281.1  B0281.3  B0281.4  B0281.5  B0281.6  B0454.5  B0454.6  B0454.8  C16C8.4  C16C8.7  C16C8.8  C16C8.9  C17F4.2  C17F4.3  C17F4.7  C41H7.2  C41H7.9  D2062.1  F02E11.3  F02E11.7  F08D12.2  F08D12.4  F08D12.7  F14D2.5  F14D2.7  F16G10.1  F16G10.2  F16G10.3  F16G10.5  F16G10.6  F16G10.7  F16G10.8  F16G10.9  F18A12.2  F18A12.7  F22E5.1  F22E5.2  F22E5.7  F22E5.8  F22E5.9  F29A7.3  F29A7.4  F33H12.1  F33H12.7  F34D6.1  F39E9.6  F42G2.2  F42G2.5  F42G2.7  F43C11.1  F43C11.2  F43C11.4  F43C11.6  F43C11.7  F43C11.8  F52C6.2  F53G2.1  F53G2.2  F53G2.3  F54A3.2  F54A3.5  F54A3.6  K02F6.1  K02F6.3  K02F6.4  K02F6.5  K02F6.7  K02F6.8  K08A2.2  K09F6.3  K09F6.7  K09F6.9  K12H6.4  K12H6.5  K12H6.6  K12H6.7  K12H6.8  K12H6.9  T05A8.1  T05A8.2  T05A8.7  T05A8.8  T06D4.1  T06D4.2  T06D4.6  T10D4.1  T10D4.6  T10D4.7  Y110A2AL.1  Y110A2AL.2  Y110A2AL.3  Y110A2AL.5  Y110A2AL.6  Y110A2AL.7  Y110A2AL.9  Y25C1A.6  Y25C1A.7  Y25C1A.8  Y27F2A.6  Y27F2A.8  Y27F2A.9  Y46D2A.1  Y46D2A.2  Y46D2A.3  Y47G7B.2  Y49F6B.2  Y49F6B.6  Y49F6B.8  Y49F6B.9  Y49F6C.2  Y49F6C.6  Y49F6C.7  Y49F6C.8  Y59C2A.3  ZC239.3  ZC239.4  ZC239.5  ZC239.6  ZK1240.1  ZK1240.2  ZK1240.3  ZK1240.5  ZK1240.6  ZK1240.8  ZK1240.9  ZK355.2  ZK355.3  ZK355.8 | *apd-3*  *bath-9*  *bath-10*  *bath-23*  *bath-27*  *bath-28*  *bath-29*  *bath-30*  *btb-16*  *btb-17*  *btb-18*  *btb-22*  *cbs-2*  *cct-3*  *ceh-86*  *ceh-87*  *chil-24*  *clec-2*  *clec-3*  *clec-121*  *clec-122*  *clec-123*  *clec-124*  *clec-125*  *clec-126*  *col-71*  *copb-1*  *cyh-1*  *enri-1*  *fbxa-4*  *fbxa-182*  *fbxb-104*  *fbxb-105*  *fbxb-106*  *fbxb-107*  *fbxb-108*  *fbxb-111*  *fbxb-112*  *fbxc-20*  *fbxc-25*  *fbxc-50*  *fbxc-55*  *fut-4*  *gbas-1*  *gcy-15*  *gcy-19*  *gcy-21*  *ins-19*  *ins-31*  *irld-27*  *irld-48*  *irld-49*  *irld-50*  *irld-63*  *irld-64*  *irld-65*  *irld-66*  *irld-67*  *lido-10*  *lido-11*  *lido-12*  *lido-13*  *lido-14*  *lido-7*  *lido-8*  *lido-9*  *lin-8*  *mnat-1*  *msrp-6*  *ncap-1*  *nep-2*  *nep-6*  *nep-8*  *nep-9*  *nep-10*  *nep-11*  *nep-13*  *nep-18*  *nep-20*  *nhr-88*  *nhr-119*  *nlp-99*  *nspf-1*  *nspf-2*  *nssp-14*  *pigw-1*  *pinn-1*  *pqn-42*  *ptc-3*  *scl-15*  *sdz-5*  *sdz-9*  *sdz-10*  *sdz-35*  *set-11*  *set-13*  *skpo-2*  *smu-2*  *sqv-2*  *srpa-72*  *sup-9*  *trim-21*  *tsr-1*  *ubc-15*  *usp-39*  *vab-19*  *wdr-31*  *wht-4* | *srg-21*  *srg-22*  *srg-23*  *srg-24*  *srh-71*  *srh-128*  *srh-297*  *sri-28*  *sri-29*  *sri-30*  *sri-31*  *sri-32*  *sri-33*  *sri-34*  *sri-36*  *sri-39*  *sri-42*  *sri-43*  *sri-48*  *sri-50*  *sri-51*  *sri-53*  *sri-54*  *sri-57*  *sri-60*  *sri-61*  *sri-62*  *sri-63*  *sri-71*  *sri-74*  *sri-77*  *srw-60*  *srz-67* |
