# Supplementary material for: Wild strains reveal natural variation in C. elegans avoidance behaviors
Source: G3 (Bethesda). 2025 Oct 10;15(12):jkaf243. doi: 10.1093/g3journal/jkaf243 (PMC12693514; doi:10.1093/g3journal/jkaf243)
Supplement: jkaf243_Supplementary_Data [file jkaf243_supplementary_data.zip › Supplemental_Table_3_G3-2025-406145.docx]

**Supplemental Table 3. Annotated genes in quinine QTL IV-R**

| Unnamed genes | Named genes  (miscellaneous) | Named genes  (predicted GPCRs) |
| --- | --- | --- |
| *C35D6.3*  *C35D6.4*  *F38C2.4*  *F38C2.7*  *H25K10.1*  *H25K10.2*  *K03D3.2*  *K03D3.5*  *VY10G11R.1*  *Y105C5A.1*  *Y105C5A.8*  *Y105C5B.1*  *Y105C5B.3*  *Y105C5B.8*  *Y10G11A.1*  *Y43D4A.5*  *Y43D4A.6*  *Y51H4A.1*  *Y51H4A.6*  *Y51H4A.7*  *Y51H4A.9*  *Y65A5A.1*  *Y65A5A.2*  *Y7A9A.1*  *Y7A9D.1* | *clec-191*  *col-137*  *daf-38*  *dlc-3*  *dlc-4*  *fip-7*  *fipr-27*  *gcy-25*  *gln-3*  *hlh-31*  *hlh-32*  *hpa-1*  *irld-39*  *jac-1*  *lgc-19*  *rac-2*  *rho-1*  *set-26*  *sta-1* | *srh-228*  *srv-11*  *srv-14*  *srv-15*  *srw-61*  *srz-38*  *srz-61*  *srz-71*  *srz-72*  *srz-74*  *srz-75*  *srz-104*  *srz-105* |
