# Supplementary figures and images for: Wild strains reveal natural variation in C. elegans avoidance behaviors
Source: G3 (Bethesda). 2025 Oct 10;15(12):jkaf243. doi: 10.1093/g3journal/jkaf243 (PMC12693514; doi:10.1093/g3journal/jkaf243)

Supplemental Figure 1

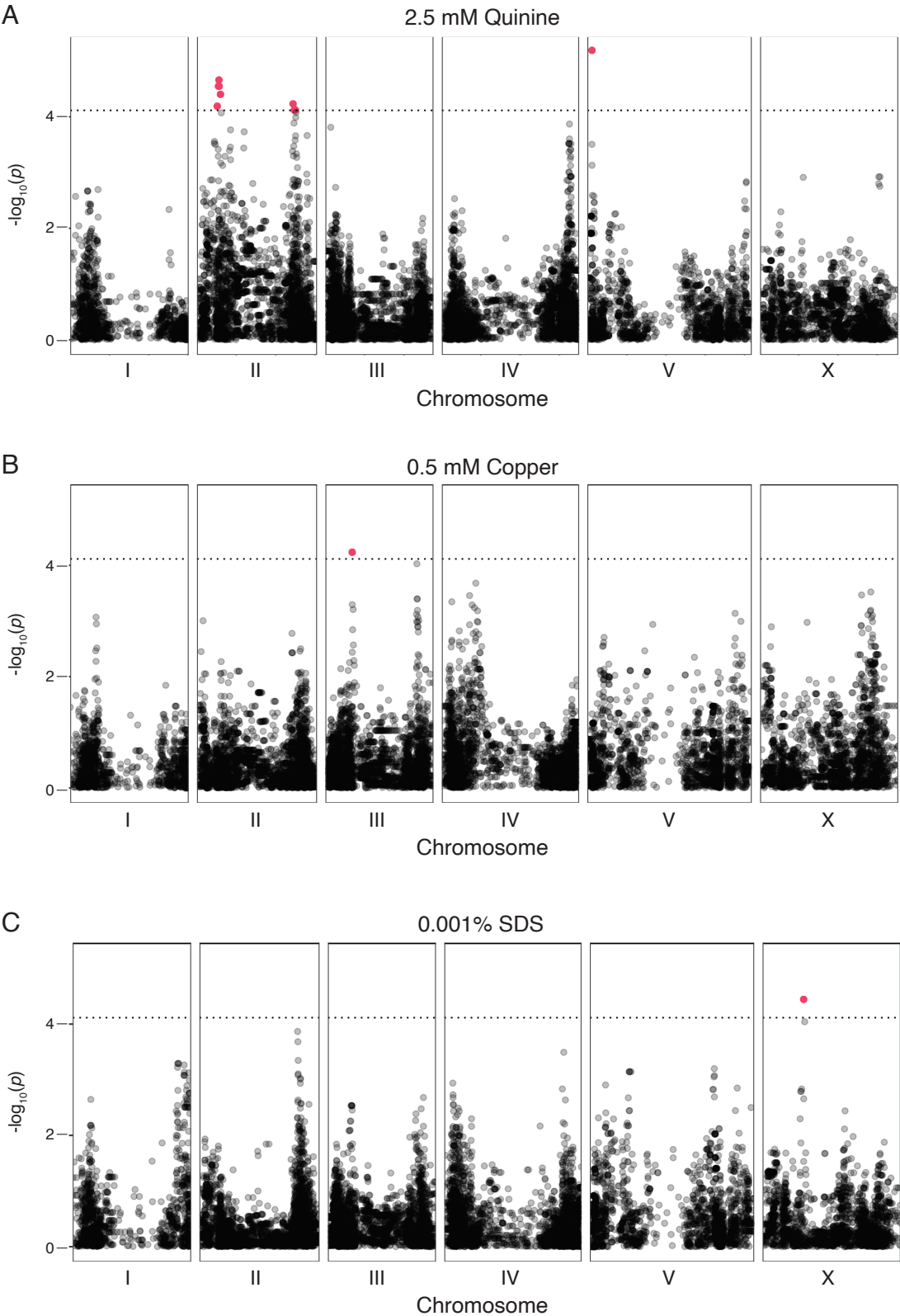

Supplement: jkaf243_Supplementary_Data [file jkaf243_supplementary_data.zip › Supplemental_Figure_1_G3-2025-406145.pdf]

Supplemental Figure 2

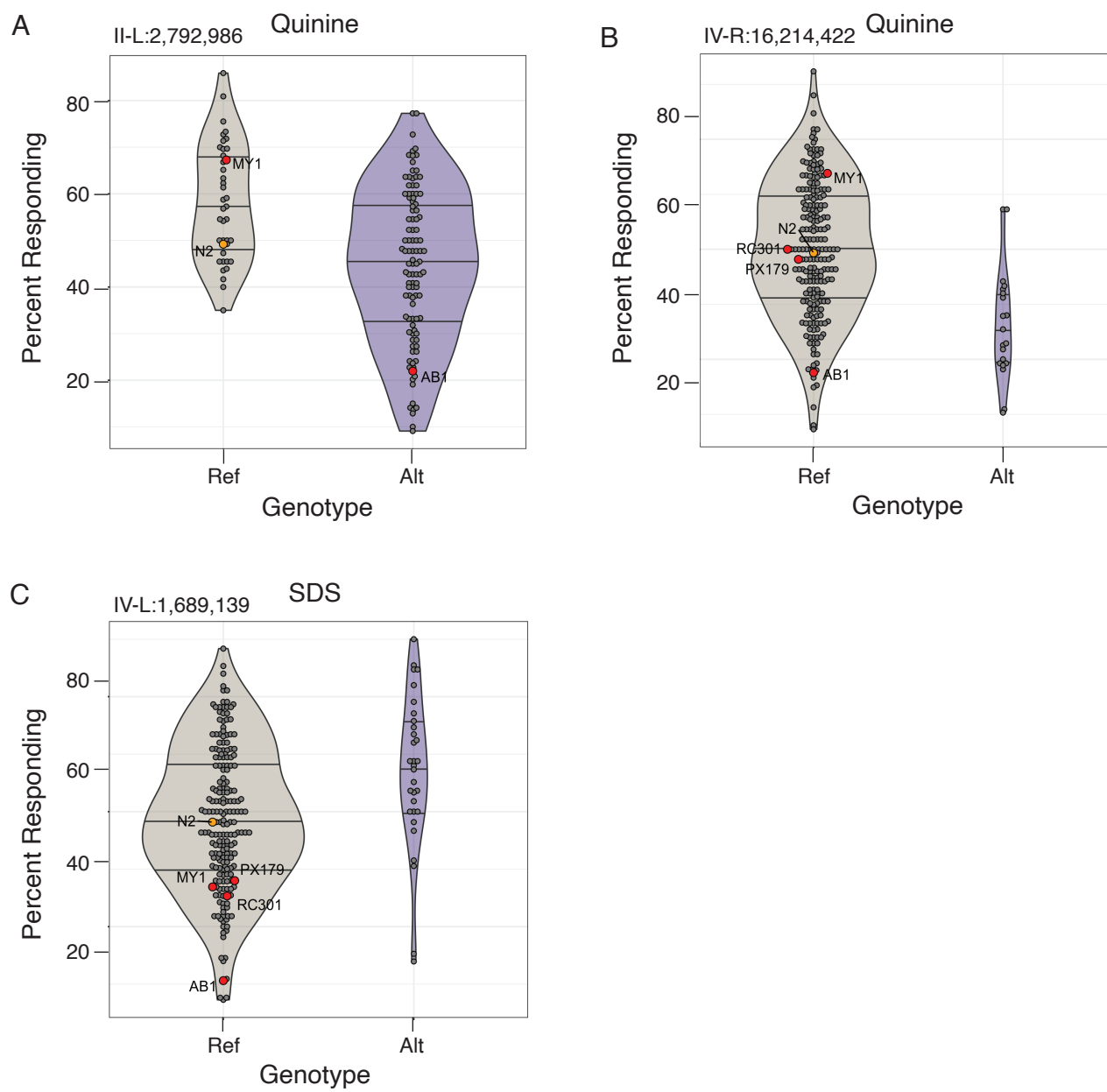

Supplement: jkaf243_Supplementary_Data [file jkaf243_supplementary_data.zip › Supplemental_Figure_2_G3-2025-406145.pdf]

Supplemental Figure 3

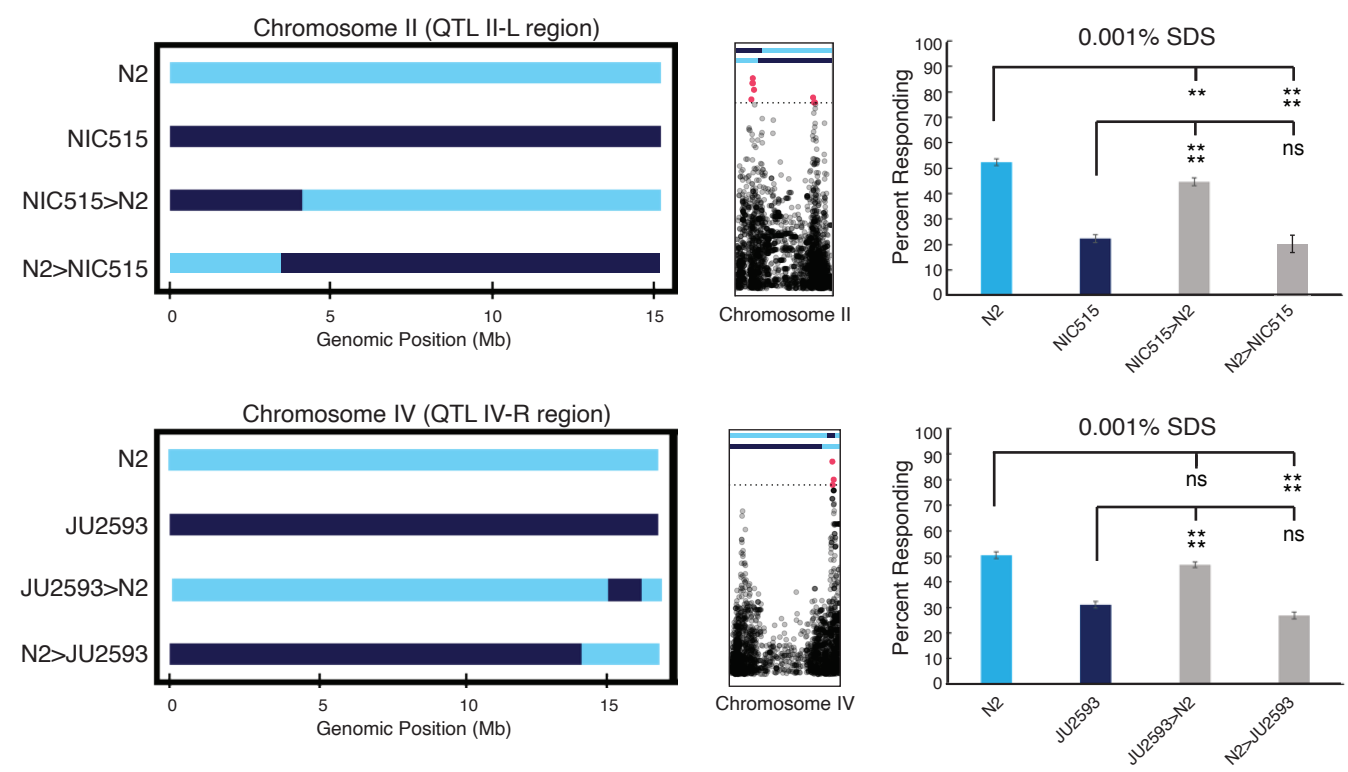

Supplement: jkaf243_Supplementary_Data [file jkaf243_supplementary_data.zip › Supplemental_Figure_3_G3-2025-406145.pdf]

Supplemental Figure 4

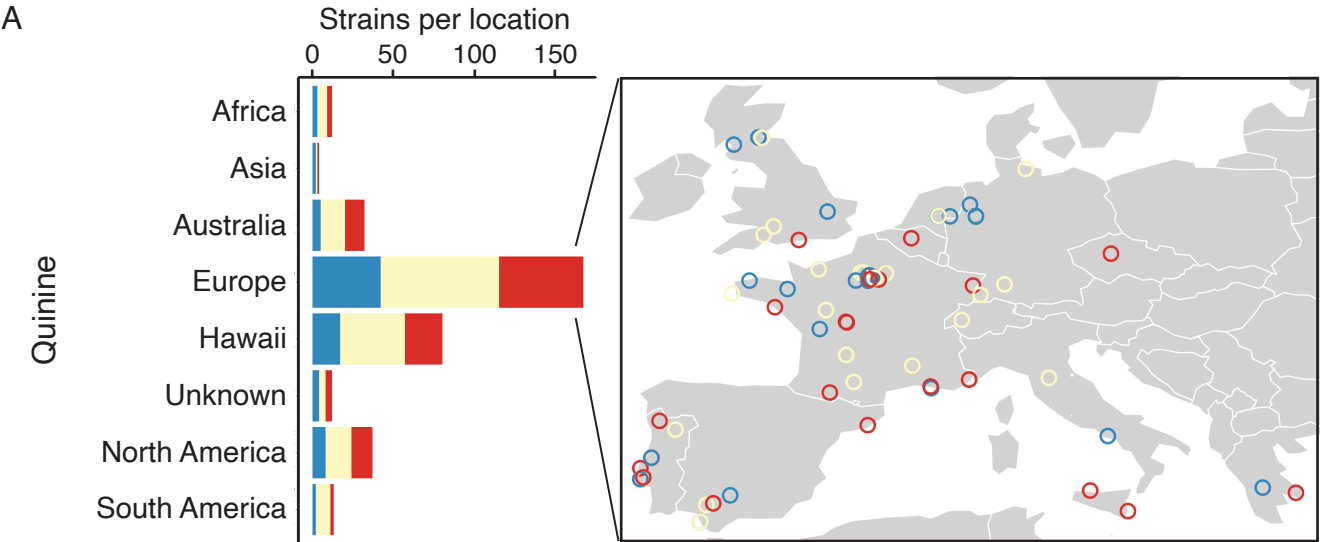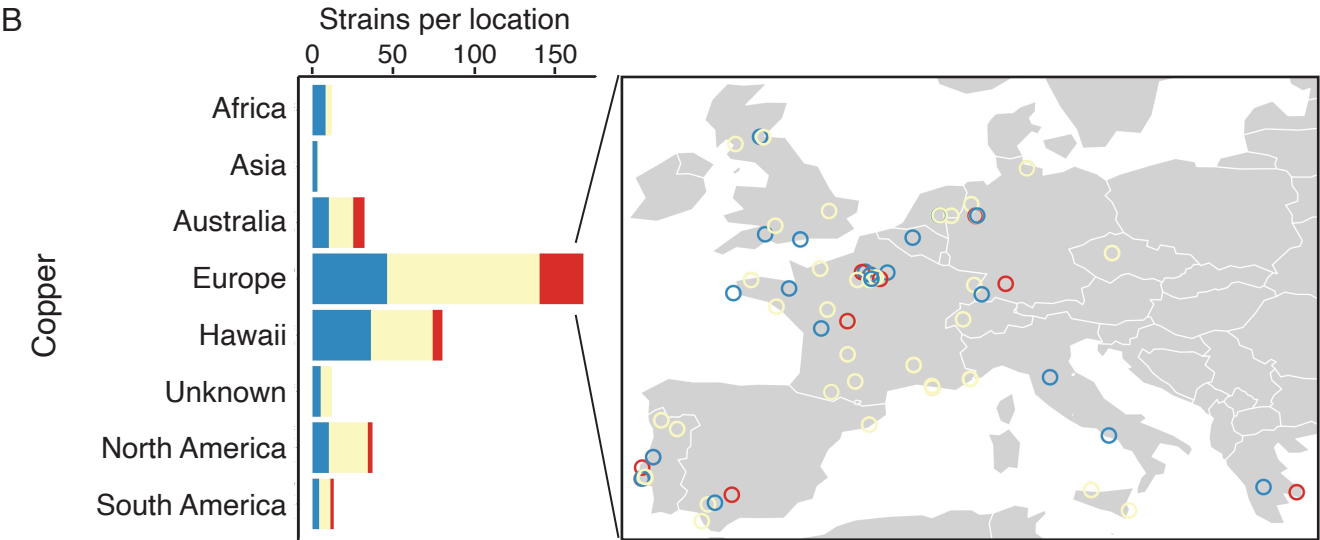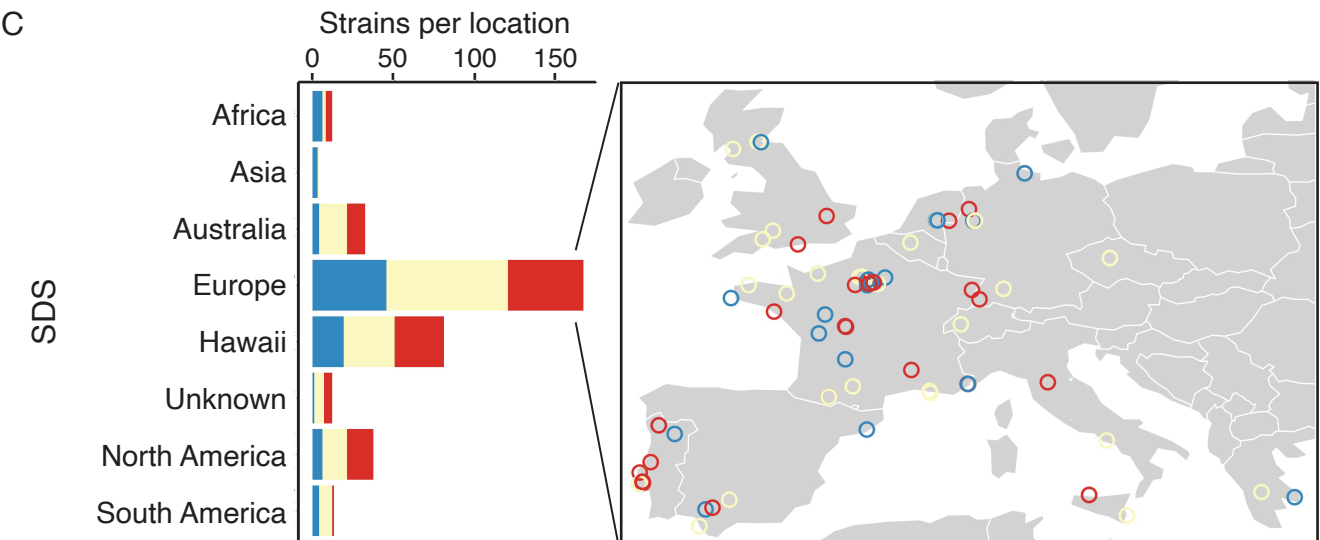

■ Hyposensitive ■ Average ■ Hypersensitive

Supplement: jkaf243_Supplementary_Data [file jkaf243_supplementary_data.zip › Supplemental_Figure_4_G3-2025-406145.pdf]
